# Assessing Patient Needs During Natural Disasters: Mixed Methods Analysis of Portal Messages Sent During Hurricane Harvey

**DOI:** 10.2196/31264

**Published:** 2021-09-01

**Authors:** Juha Baek, Bridget Simon-Friedt, Adriana Lopez, Jacob M Kolman, Juan Nicolas, Stephen L Jones, Robert A Phillips, Terri Menser

**Affiliations:** 1 Center for Outcomes Research Houston Methodist Houston, TX United States; 2 Department of Surgery Houston Methodist Houston, TX United States; 3 Department of Surgery Weill Cornell Medical College New York, NY United States; 4 Department of Cardiology Houston Methodist Houston, TX United States; 5 Department of Medicine Weill Cornell Medical College New York, NY United States; 6 Department of Population Health Sciences Weill Cornell Medical College New York, NY United States

**Keywords:** Hurricane Harvey, natural disasters, patient portals, electronic messages, emergency preparedness, disaster medicine

## Abstract

**Background:**

Patient portals play an important role in connecting patients with their medical care team, which improves patient engagement in treatment plans, decreases unnecessary visits, and reduces costs. During natural disasters, patients’ needs increase, whereas available resources, specifically access to care, become limited.

**Objective:**

This study aims to examine patients’ health needs during a natural crisis by analyzing the electronic messages sent during Hurricane Harvey to guide future disaster planning efforts.

**Methods:**

We explored patient portal use data from a large Greater Houston area health care system focusing on the initial week of the Hurricane Harvey disaster, beginning with the date of landfall, August 25, 2017, to August 31, 2017. A mixed methods approach was used to assess patients’ immediate health needs and concerns during the disruption of access to routine and emergent medical care. Quantitative analysis used logistic regression models to assess the predictive characteristics of patients using the portal during Hurricane Harvey. This study also included encounters by type (emergency, inpatient, observation, outpatient, and outpatient surgery) and time (before, during, and after Hurricane Harvey). For qualitative analysis, the content of these messages was examined using the constant comparative method to identify emerging themes found within the message texts.

**Results:**

Out of a total of 557,024 patients, 4079 (0.73%) sent a message during Hurricane Harvey, whereas 31,737 (5.69%) used the portal. Age, sex, race, and ethnicity were predictive factors for using the portal and sending a message during the natural disaster. We found that prior use of the patient portal increased the likelihood of portal use during Hurricane Harvey (odds ratio 13.688, 95% CI 12.929-14.491) and of sending a portal message during the disaster (odds ratio 14.172, 95% CI 11.879-16.907). Having an encounter 4 weeks before or after Hurricane Harvey was positively associated with increased use of the portal and sending a portal message. Patients with encounters during the main Hurricane Harvey week had a higher increased likelihood of portal use across all five encounter types. Qualitative themes included: access, prescription requests, medical advice (chronic conditions, acute care, urgent needs, and Hurricane Harvey–related injuries), mental health, technical difficulties, and provider constraints.

**Conclusions:**

The patient portal can be a useful tool for communication between patients and providers to address the urgent needs and concerns of patients as a natural disaster unfolds. This was the first known study to include encounter data to understand portal use compared with care provisioning. Prior use was predictive of both portal use and message sending during Hurricane Harvey. These findings could inform the types of demands that may arise in future disaster situations and can serve as the first step in intentionally optimizing patient portal usability for emergency health care management during natural disasters.

## Introduction

The prevalence of natural disasters such as storms and severe flooding has increased over the last century, both nationally and globally, leading to substantial human and financial loss [1-3]. In the United States, the average number of disasters per year has significantly risen from 1.63 events per year (from 1917 to 1966) to 14 events per year (from 1967 to 2016) [3]. Since the early 1990s, the average annual number of disasters has dramatically escalated to 19.7 events [3]. Of the 10 worst natural catastrophes in US history, 9 occurred after 2000.

Hurricane Harvey was a Category 4 hurricane that made landfall along the Texas coast on August 25, 2017. This storm deposited more than 60 inches of rain onto the city of Houston and surrounding areas and caused more than 100 deaths because of direct or indirect effects [4]. It also adversely affected approximately 200,000 homes, causing an estimated US $125 billion in damage, making it the second most financially devastating storm after Hurricane Katrina in 2005 [5]. The resultant flooding and damage caused by Harvey brought health care access challenges for area residents across the Greater Houston community. Other recent disasters include the COVID-19 pandemic and winter storm Uri (February 2021) that shut down the Houston area for 5 days and caused loss of life because of infrastructure inadequacies. Both disasters affected access to traditional health care settings. The operational lessons learned during Hurricane Harvey have been previously described and summarize the organizational response during natural disasters [6]. During such disasters, the medical needs of patients increase, whereas available resources become limited. As a result of such emergency situations, health care infrastructures are often unable to operate normally, thus restricting patients’ access to timely care and communication with their providers [7].

Digital patient portals were first introduced in the late 1990s and are defined as secure web-based tools that allow patients to access their health information and communicate with their health care team through electronic messages [8-11]. Health care organizations received government incentives under the Meaningful Use Incentive Program of the Health Information Technology for Economic and Clinical Health Act of 2009 which has increased the adoption and use of patient portals in health care [12-14]. Current statistics suggest that approximately 90% of the health care organizations provide some type of portals to their patients, and a 2016 American Hospital Association report also showed that approximately 92% of the patients are able to access their health records [15,16]. Patient portals contribute to improving patient engagement in health care, facilitating patient-centered care, enhancing satisfaction with care, reducing unnecessary visits and phone calls, and decreasing health care service use and costs [8,17-21].

Patient portals have a wide array of functionalities of potential relevance in a disaster scenario, including secure messaging between patients and health care providers; access to medical records, test results, and education resources; and medication refills [22-24]. In addition, portal access is often a prerequisite to being able to schedule and attend telehealth visits, which have increased significantly since the COVID-19 pandemic struck [25-27]. Secure messaging has been reported to be among the most commonly used features of patient portals [23,24], and qualitative assessment of patient portal message content has been conducted across various patient populations, including patients with diabetes, surgical patients, and patients in the primary care setting [10,28,29]. Prior studies have shown that most messages were related to patients’ medical needs such as appointments, medical problems, and test-related content [28,29].

Although previous studies have examined portal messages sent between patients and providers across various patient populations and health care settings, these evaluations were conducted during periods of standard care. Given that patients’ needs increase during emergency situations, communication between patients and providers through patient portals could be an effective tool to help patients manage their health status and improve health outcomes in a period of heightened medical need. However, to our knowledge, no study has evaluated patients’ electronic messages or patient portal use during a disaster. Therefore, this study aims to examine patients’ health needs during a natural crisis, which can help guide future intentional efforts with regard to using patient portals during disaster periods.

## Methods

### Design and Setting

This study was conducted at Houston Methodist, which is a large health system in the Greater Houston area comprising 8 hospitals, including an academic hospital. All patients can request access to the web-based patient portal, which allows them to communicate with health care providers, check laboratory test results, update medications, request appointments, and manage their care on the web. This study used a mixed methods approach to assess patients’ immediate health needs during an emergency situation resulting from Hurricane Harvey, which was defined in this study as the day of hurricane landfall and the following 6 days (from August 25, 2017, to August 31, 2017).

The study population was limited to patients aged 18 to 105 years, and portal access and portal nonaccess were compared by demographic characteristics. We included the entire electronic health record (EHR) population for 2017 and created categories for missing demographic information instead of omitting these individuals. Omitting patients for selecting the *prefer not to answer* response for race and ethnicity, specifically, could have unnecessarily decreased the total population and unintentionally reduced its diversity. In addition, all patients in the quantitative model have data for portal activity during the week of Hurricane Harvey, which is the main study objective. This study was reviewed and approved by the institutional review board of the Houston Methodist Research Institute.

### Measures

#### Outcomes

Portal users were distinguished from the total patient population in 2017 using a binary indicator for those who had an active portal account, regardless of use. Portal use was measured using patient-level audit trail data obtained from the EHR and was coded binarily; Harvey portal use was defined as at least one log-in during the designated disaster period week from August 25, 2017, to August 31, 2017. Harvey message sending was also coded in a binary manner, with *1* assigned to all users who sent at least one message during the study period.

#### Covariates

The main independent variable was the binary use of the patient portal before Harvey in 2017, defined as a single log-in between January 1, 2017, and August 24, 2017. The demographic factors included age (18-44 years, 45-64 years, ≥65 years, or unknown), sex (female or male), and race (White, Black, Asian, Other, or Unknown). The Other category collapsed races with small sample sizes and included Native Hawaiian or Other Pacific Islander and American Indian or Alaska Native. The Unknown category included patients who declined to answer demographic questions or EHRs with no value for the respective category. Binary measures of a defined set of comorbidities using the Charlson Comorbidity Index [30] were created using patient-level *International Classification of Diseases, Tenth Revision*, codes based on discharge dispositions that accompany all encounter types.

In all, five types of encounter variables were constructed based on the date of appointment and included emergency department (ED) visits, inpatient stays, observations, outpatient visits, and surgeries. These encounter types were selected because they were five of the most common encounter types of a total of 23 encounter types that year, representing 87% of the total encounters. The categories for each of these encounter variables were developed by considering the patients’ likelihood of or need for interaction with the health care system, which we assumed would be relative to their receipt of care related to the disaster timeline (eg, patients’ portal use would likely increase just before or directly after an appointment, procedure, or other encounter). The encounter categories were defined as (1) no encounter in 2017; (2) an encounter in 2017, excluding the 4 weeks before, the week of, and the 4 weeks after Hurricane Harvey (January 1-July 27, 2017, or September 29-December 31, 2017); (3) the 4-week period both immediately before (July 28-August 24, 2017) and immediately after the week of Harvey (September 1-September 28, 2017); and (4) an encounter during the week of Harvey (August 25-August 31, 2017). 

### Analytic Methods

The quantitative evaluation included two main binary indicators of portal use: portal use of any kind and message sending during the defined study period. The qualitative evaluation component identified messages by date and examined all message content sent through the patient portal during the week of Hurricane Harvey. Further quantitative analyses focused on portal use were limited to the subset of patients with a portal account because access to the portal was a prerequisite.

Descriptive statistics were calculated for the total patient population in 2017 and patients with an activated portal account and compared with those of portal nonusers, in addition to the subpopulations of interest in this study (ie, portal use and message sending during Harvey), to readily allow for comparison of the patients’ characteristics. The mean and SD were calculated for continuous variables, and frequencies and percentages were used for categorical variables. We used two-tailed *t* tests or Pearson chi-square tests to initially examine differences between portal users and portal nonusers but presented standardized mean differences (SMDs) given the large sample size of the study population; an absolute value greater than or equal to 0.10 of the SMD was considered statistically significant [31]. We used logistic regression models to assess predictors of portal use (model 1) and portal message use (model 2) during Hurricane Harvey. All statistical analyses were conducted using Stata version 16 (StataCorp LLC). Statistical significance was set at *P*<.05.

### Qualitative Methods

All electronic messages sent by patients through the web-based patient portal during the study period were extracted from the EHR, and message IDs were used to pair individual messages with the sender and receiver. Structured query language was used to format the message threads and export them to a spreadsheet. All automated or systemwide messages were excluded, such as bulk messages regarding office closures and other disaster-related communications sent from the health system. Initially, we attempted to segregate Harvey-related messages from standard care messages, but given the subjective nature of defining a Harvey-related message, all messages from the study period were reviewed in the qualitative thematic analysis [32,33]. The first message file (4354/9316, 46.74% of messages) was coded without a priori defined themes by JB, BSF, and TM using the constant comparative method, which is an inductive data coding process [34]. After reviewing the common themes across these initial messages, the following targeted research questions were developed:

1. *What problems did patients face during Harvey that they communicated through the patient portal?*

2. *How were providers able to help patients through the patient portal?*

3. *What needs were providers unable to address through the patient portal?*

The coding of the remaining messages (4962/9316, 53.26%) was guided by these questions and performed using Quirkos software version 2.4.1 (Quirkos) [35]. Both initial coding rounds were combined into a Microsoft Excel (Microsoft Corporation) file, where the second-level theme assignment was carried out individually by JB and BSF. Weekly group meetings, which included TM, JB, BSF, and JMK, with other authors attending as needed, were held to discuss and condense second-level themes. The codes created by the 2 primary coders (JB and BSF) were compared during these meetings, and inconsistencies between them were resolved through discussion to reach a consensus.

## Results

### Overview

**Figure 1:**
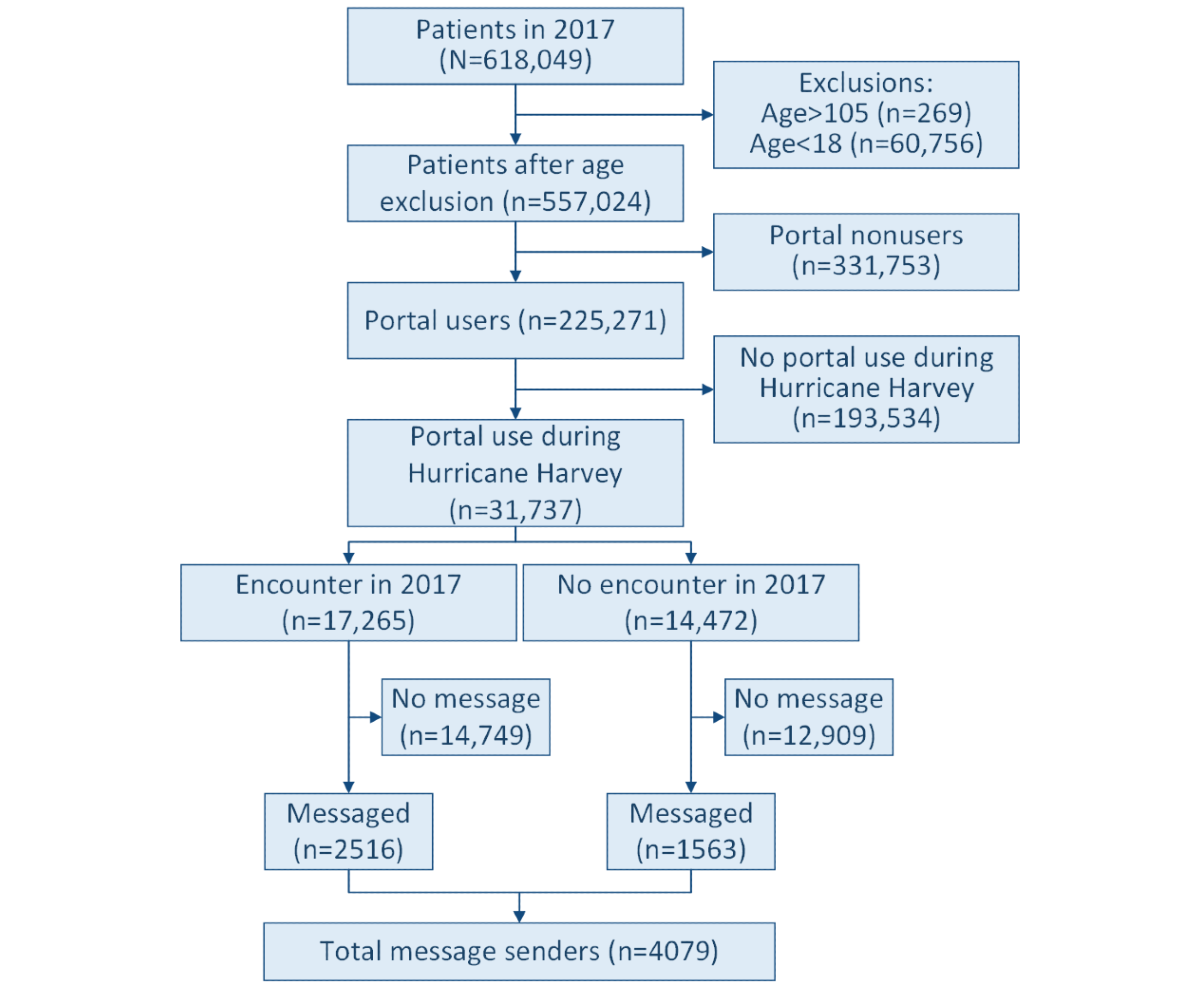
Flow diagram of selection process for study population and total number of patients who messaged during Hurricane Harvey.

The study sample included 557,024 patients aged between 18 and 105 years. We categorized this population into portal users, portal nonusers, portal users during Harvey, and message senders during Harvey, in addition to categorizing portal users with and without encounters in 2017 (Figure 1). The percentage of patients with an activated portal account (portal users) and those with no account (portal nonusers) was 45.82% (255,271/557,024) and 59.55% (331,753/557,024), respectively. Among portal users in 2017, 12.43% (31,737/255,271) used the portal during Harvey, which is 5.69% (31,737/557,024) of the total Houston Methodist patient population. It is interesting to note that of these patients, 54.4% (17,265/31,737; encounter) had at least one appointment in 2017, and 24.63% (7816/31,737) of these patients had an appointment surrounding (4 weeks before or 4 weeks after) or during the week of Harvey. Of the 4079 patients who sent a portal message during Harvey, 2516 (61.68%) had an appointment in 2017 and 1317 (32.28%) had an appointment either 4 weeks before, the week of, or 4 weeks after Harvey.

A total of 11.96% unique patients (66,647/557,024 of the total patients) sent a portal message at least once in 2017, totaling 364,617 messages, with an average of 1.62 messages per patient. Among these messages, approximately 2.55% (9316/364,617) were sent during Harvey, with an average of 2.3 messages per patient. Analysis of the number of messages sent per day showed that the most number of messages were sent on Thursday (the last day) during the week of Harvey (2981/9316, 31.99%), followed by Wednesday (the sixth day: 1981/9316, 21.26%) and Friday (the first day: 1726/9316, 18.53%). Overall, we found that although patient portal use declined during Harvey, portal messaging maintained near average patient-level use. In 2017, 13.8% of unique patients of the total activated users used the messaging function of the portal [32] compared with 12.85% (4079/31,737) of the patients who logged into the portal during Harvey and who also sent a message.

### Quantitative Results

#### Portal Access and Portal Use During Harvey

Multimedia Appendix 1 shows the descriptive statistics of the total number of patients in 2017 (n=557,024) and of the subpopulations of interest: patients who used the portal during Harvey (n=31,737) and patients who messaged during Harvey (n=4079). Most of the patients who messaged and used the portal were aged 45 years or older, female, White, and non-Hispanic/Latinx. Of the total number of portal users during Harvey, 43.51% (13,808/31,737) had an outpatient encounter, as did more than half (2065/4079, 50.62%) of the patients who messaged during Harvey, which is consistent with the number of overall portal users in 2017 because patients with outpatient encounters composed the largest percentage of portal users in general. When comparing portal users and portal nonusers in 2017, we found that portal users were more likely to be aged below 65 years, female, White, and non-Hispanic/Latinx compared with portal nonusers. Age, race, and ethnicity were statistically significant different between the portal user groups (SMD >0.10), although sex was not. The patients who used the portal in 2017 had lower percentages of most of the known comorbidities except cancer, connective tissue disease, and mild liver disease compared with their counterparts; however, SMDs for all comorbidities were less than 0.10.

#### Portal Use During Hurricane Harvey

To examine the predictors of patient portal use during Hurricane Harvey, we used multivariate logistic regression (model 1; Table 1). We found that the patients who had used the portal before Harvey in 2017 were approximately 13.7 times more likely to use the portal during Harvey than those who had not used the portal (odds ratio [OR] 13.688, 95% CI 12.929-14.491; *P*<.001). Compared with the patients aged 18-44 years, those aged 65 years or older were 1.13 times as likely to use the portal during Harvey (OR 1.128, 95% CI 1.091-1.166; *P*<.001), but those aged 45-64 years were less likely to use the portal during Harvey (OR 0.949, 95% CI 0.921-0.978; *P*=.001). Male and Hispanic/Latinx patients were less likely to use the portal during Harvey than female and non-Hispanic/Latinx patients (OR 0.963, 95% CI 0.938-0.989, *P*=.005 and OR 0.860, 95% CI 0.823-0.898, *P*<.001, respectively). The results also showed that patients who had an appointment within 4 weeks before or after Harvey were more likely to use the portal during Harvey than those who did not have any appointment in all types of encounters (OR 2.052, 95% CI 1.973-2.134 for outpatient; OR 1.810, 95% CI 1.681-1.949 for surgery; OR 1.608, 95% CI 1.444-1.790 for observations; and OR 1.673, 95% CI 1.551-1.804 for inpatient, *P*<.001 and OR 1.085, 95% CI 1.001-1.175 for ED, *P*=.046). In addition, the positive associations between having an appointment during Harvey and using the portal during Harvey were found to be stronger for each type of encounter than with having an appointment within 4 weeks before or after Harvey (OR 5.897, 95% CI 5.094-6.826 for outpatient; OR 4.337, 95% CI 3.106-6.057 for surgery; OR 4.697, 95% CI 3.366-6.554 for observations; OR 2.917, 95% CI 2.340-3.635 for ED; and OR 2.743, 95% CI 2.307-3.261 for inpatient; *P*<.001).

**Table 1:** Results of logistic regression models fitted to the outcomes: used the patient portal during Harvey (Model 1) and messaged during Harvey (Model 2).

Independent variables		Model 1: used patient portal during Harvey		Model 2: messaged during Harvey	
		OR^a^ (95% CI)	*P* value	OR (95% CI)	*P* value
**Used portal before Harvey in 2017 (reference: no portal use in before Harvey in 2017)**					
	Yes	13.688 (12.929-14.491)	<.001	14.172 (11.879-16.907)	<.001
**Age (years;** **reference: 18-44 years)**					
	45-64	0.949 (0.921-0.978)	.001	1.168 (1.081-1.262)	<.001
	≥65	1.128 (1.091-1.166)	<.001	1.298 (1.192-1.413)	<.001
**Sex** **(reference: female)**					
	Male	0.963 (0.938-0.989)	.005	0.899 (0.841-0.962)	.002
**Race** **(reference: White)**					
	Black	0.977 (0.940-1.017)	.26	0.859 (0.775-0.952)	.004
	Asian	0.987 (0.939-1.038)	.61	0.793 (0.690-0.912)	.001
	Other	1.097 (1.031-1.168)	.004	1.081 (0.919-1.272)	.34
	Unknown	0.990 (0.929-1.056)	.76	1.015 (0.859-1.198)	.86
**Ethnicity** **(reference: non-Hispanic/Latinx)**					
	Hispanic/Latinx	0.860 (0.823-0.898)	<.001	0.854 (0.763-0.955)	.006
	Unknown	0.978 (0.900-1.061)	.59	1.008 (0.814-1.248)	.94
**Outpatient** **(reference: no encounter in 2017)**					
	Encounter in 2017 outside Harvey period^b^	0.898 (0.872-0.925)	<.001	1.037 (0.961-1.120)	.35
	Encounter peri-Harvey^b^	2.052 (1.973-2.134)	<.001	2.465 (2.260-2.689)	<.001
	Encounter during Harvey^b^	5.897 (5.094-6.826)	<.001	5.009 (3.944-6.362)	<.001
**Surgery (reference: no encounter in 2017)**					
	Encounter in 2017 outside Harvey period^b^	0.953 (0.912-0.996)	.03	0.989 (0.890-1.100)	.85
	Encounter peri-Harvey^b^	1.810 (1.681-1.949)	<.001	1.762 (1.514-2.050)	<.001
	Encounter during Harvey^b^	4.337 (3.106-6.057)	<.001	0.583 (0.184-1.843)	.36
**Observations** **(reference: no encounter in 2017)**					
	Encounter in 2017 outside Harvey period^b^	0.861 (0.810-0.915)	<.001	0.931 (0.807-1.075)	.33
	Encounter peri-Harvey^b^	1.608 (1.444-1.790)	<.001	1.444 (1.156-1.802)	<.001
	Encounter during Harvey^b^	4.697 (3.366-6.554)	<.001	1.201 (0.549-2.628)	.65
**Emergency department** **(reference: no encounter in 2017)**					
	Encounter in 2017 outside Harvey period^b^	0.722 (0.690-0.755)	<.001	0.900 (0.809-1.002)	.06
	Encounter peri-Harvey^b^	1.085 (1.001-1.175)	.046	1.407 (1.188-1.667)	<.001
	Encounter during Harvey^b^	2.917 (2.340-3.635)	<.001	2.317 (1.505-3.565)	<.001
**Inpatient** **(reference: no encounter in 2017)**					
	Encounter in 2017 outside Harvey period^b^	0.903 (0.861-0.947)	<.001	1.039 (0.930-1.161)	.50
	Encounter peri-Harvey^b^	1.673 (1.551-1.804)	<.001	1.882 (1.619-2.186)	<.001
	Encounter during Harvey^b^	2.743 (2.307-3.261)	<.001	2.088 (1.498-2.909)	<.001

^a^OR: odds ratio.

^b^Outside Harvey period defined as the periods January 1-July 27, 2017, and September 29-December 31, 2017; peri-Harvey period defined as the 4-week periods both immediately before Harvey (July 28-August 24, 2017) and immediately after Harvey (September 1-September 28, 2017); and during Harvey period defined as August 25-August 31, 2017.

#### Message Sending During Hurricane Harvey

To explore the factors associated with patients sending a message during Harvey, we also used multivariate logistic regression analysis (model 2; Table 1). The results revealed that prior portal use in 2017 was positively associated with sending a message during Harvey (OR 14.172, 95% CI 11.879-16.907; *P*<.001). Patients aged 45-64 years or those aged 65 years or older were more likely to send a message through the portal during Harvey than those aged 18-44 years (OR 1.168, 95% CI 1.081-1.262 for those aged 45-64 years and OR 1.298, 95% CI 1.192-1.413 for those aged 65 years or older; *P*<.001). Male, Black, Asian, and Hispanic/Latinx patients were less likely to send a portal message during Harvey than their counterparts (OR 0.899, 95% CI 0.841-0.962, *P*=.002 for sex; OR 0.859, 95% CI 0.775-0.952, *P*=.004 for Black patients; OR 0.793, 95% CI 0.690-0.912, *P*=.001 for Asian patients; OR 0.854, 95% CI 0.763-0.955, *P*=.006 for Hispanic/Latinx patients). We found that patients with an appointment within 4 weeks before or 4 weeks after Harvey were more likely to send a portal message during Harvey than those without an encounter for each type of encounters (OR 2.465, 95% CI 2.260-2.689 for outpatient; OR 1.762, 95% CI 1.514-2.050 for surgery; OR 1.444, 95% CI 1.156-1.802 for observations; OR 1.407, 95% CI 1.188-1.667 for ED; and OR 1.882, 95% CI 1.619-2.186 for inpatient; *P*<.001). Furthermore, patients who had outpatient, ED, or inpatient encounters during Harvey showed higher ORs for sending a message during Harvey than those who had no encounter (OR 5.009, 95% CI 3.944-6.362 for outpatient; OR 2.317, 95% CI 1.505-3.565 for ED; and OR 2.088, 95% CI 1.498-2.909 for inpatient; *P*<.001).

### Qualitative Results

#### Overview

A total of 9316 electronic messages (inclusive of sent and received messages) were paired with 4079 unique patients during the study period and evaluated to identify emergent themes. Despite electricity outages during Hurricane Harvey, portal users were still able to communicate with providers during this disruption of care, although portal use decreased overall during this period compared with the rest of the year. We categorized message content into the following themes that summarize patients’ needs and providers’ responses during Hurricane Harvey: access, prescription requests, medical advice, mental health, technical difficulties, and provider constraints.

#### Access

Messages related to access included no *physical access to health care* (eg, physician office closures requiring rescheduling or rerouting), no *physical access to pharmacy services* (eg, pharmacy closures due to flooding and power outages), *structural access issues* (eg, roadway conditions or flooding blocking transportation to health services), and patients’ *inability to access established health services* (eg, evacuation to a nonflooded area or being stranded away from home). Sometimes, these varied types of access would overlap; for example, a displaced patient unable to access their established surgical team described a structural access issue despite having physical access that required rescheduling:

Due to the weather they called me yesterday to cancel my preop appointment, but they kept my surgery for tomorrow. Unfortunately, there is NO WAY I can get out of my house tomorrow, the water on the street is too high to allow us to drive to the hospital...I need to reschedule all my appointments.

Portal user

Several patients requested a web-based referral because the disaster required them to temporarily relocate to another city and cancel existing appointments; however, the serious nature of the condition required ongoing specialized care.

My wife and I fortunately relocated to [city name] just before Harvey as it turns out and now are only dealing with issues regarding our house in Houston which was flooded. Since I have a need for a cardiologist in [city], could you kindly refer me to one?

Portal user

In addition, patients sought direction from providers, exchanging messages on when and how to safely reach health facilities. Patients sometimes asked for suggestions on where to find accessible pharmacy locations, and providers routinely resent prescriptions and laboratory test orders to different locations to enable patient access:

Script is on the way! Hope the pharmacy is open! Let me know either way. We are trying to assess the area. All the roads out of [location] are blocked by water.

Provider

Patients and providers used the portal to discuss and confirm appointments while requesting or providing advice on navigating the roadways:

Do you know about [location] area?...I’ll just try to keep my appointment for tomorrow...

Portal user

249 and 1960 are open and I’m here today if you want to come in this morning. Let me know.

Provider

#### Prescription Requests

Patients frequently messaged providers regarding prescription needs due to *their inability to acquire their medications*, fear of *running out during the disaster period* (eg, prescription refills), and *emerging medical needs* that resulted from the storm (eg, new prescription requests or replacement medications). Most patients asked to refill prescriptions because they were running out of medication. Some patients described situations in which their medications were destroyed or unreachable because of flooding and requested a prescription refill:

My prescriptions along with everything in my house have been destroyed. My whole house is under water and I haven’t gotten to take any of my medicine...please put in another prescription to me for [pharmacy name]? I haven’t even taken one single pill out of the medicine you gave me.

Portal user

Several messages came from patients who could not return because of Harvey but needed to acquire the medication out of state:

I am currently stranded outside of Boston, MA, and we’re not sure when we’ll make it back. I have two more days of medication. Is there any way you can send in an emergency prescription to [pharmacy name] at [address in MA]?

Portal user

Other patients messaged that they were struggling to get the medication because of weather-related pharmacy closures or lack of medication available at their local pharmacy. They asked providers to resend a prescription to an alternative available pharmacy, saying as follows:

The pharmacist assistant at [pharmacy name] on [pharmacy location road] informed me that the prescription that Dr.[X] called in for me for [beta blockers] is difficult to get...Can Dr. [X] prescribe something else for me?

Portal user

Some messages included context related to delay or disruption of care due to difficulties in acquiring medication from a pharmacy or a delayed prescription approval from a provider during the hurricane situation:

He took the last of his [immunomodulatory drug] on Aug. 22...However, the pharmacy notified us that because of the storm the medication could not be delivered...As of today, we have not received his prescription.

Portal user

#### Medical Advice

A varied heterogeneous family of portal message topics reflected patients’ need for medical advice during the disaster. We found that the types of medical advice that patients were seeking were related to *chronic conditions*, *acute care*, *urgent needs*, and *Harvey-related injuries*.

Advice was sought during the disaster for existing *chronic health conditions*, including diabetes, hypertension, seizures, and migraines. The questions included medication adjustments, symptom assessment, and management until regular health care operations could resume. A patient messaged during the night with concern regarding elevated hypertension and associated physical symptoms. The patient described severe headaches, facial numbness, and dizziness and used the portal to seek medical advice after avoiding a potentially crowded medical facility:

I need your advise how [sic] to manage my [hypertension] at night...I was told Methodist WB is so packed and people were waiting to be seen I elected not to go, I was able to sleep for 3 hrs and this morning my bp was 146/90, Can I take [antihypertension drug] 50 mg in am and [Calcium channel blocker] 10 mg at night...

Portal user

Patients used the portal to seek medical advice for *acute illnesses* that occurred during the disaster period. These messages detailed symptoms of common ailments, including allergies, urinary tract infections, and upper respiratory infections. Providers were able to readily address many of these acute care needs through the portal:

I came home with a wicked sore throat. It hurts when I talk and swallow. I get strep all the time and I’m worried about that. I checked with a flashlight and it looks like there is white in the back of my throat. I am not 100% sure. I’m worried about driving in, and worried you won’t have an appointment. If I can’t get into see you what should I do?

Portal user

Hi [name], I am sending amoxicillin to your [pharmacy name] now.

Provider

There were instances in which patients reached out to their providers for *urgent guidance,* including patients who had recently undergone surgery in the weeks before the hurricane who messaged for postsurgery guidance. In addition, pregnant patients nearing anticipated delivery dates used the portal to reach their providers to describe new symptoms that could affect their pregnancy and to make alternative birthing plans if delivery were to occur during the disaster or if they were displaced because of evacuation*.* An urgent message request was sent by a pregnant patient to obtain guidance and to transfer her patient information:

I’m leaking some sort of fluid can a nurse call me [phone number]...Can you send over my records to the hospital I’m in now...

Portal user

Other patients sought advice regarding Hurricane *Harvey–related injuries*. The portal reflected specific questions about injuries resulting from disaster preparations to protect property (eg, strained muscles from moving furniture) as well as postdisaster recovery efforts, including exposure to unclean flood water. These types of messages included concerns related to infection and tetanus through minor injuries:

...are you opened [sic] today? I got a cut on my leg while helping rescuing on Monday. I spoke with Dr [name] and he said I need to be seen and need a tetanus sho[t]...

Portal user

Other patients sought medical advice after a substantial injury and the inability to receive routine medical care during the disaster:

Dr [name], during the flood I slipped in my house and believe I have broken at least one toe...My foot continues to swell and 3 toes are bruised...Would you be able to just send an order for an xray to San Jacinto outpatient bldg for xray? The...[m]edical clinic close to my house has not been open all wee[k]...

Portal user

#### Mental Health

Several patients expressed feelings of experiencing tragedy, anxiety, and depression in relation to the storm, flooding, and loss of property. Patients used words such as “devastated” and “surreal” to convey their feelings to health care providers. These messages were often sent as prescription requests for short-term medication options to aid in coping during the disaster. Portal users described physical symptoms associated with the mental health aspects of storm-related anxiety, especially the inability to sleep:

We had flooding in our house, and it’s been stressful. I’m wondering if you could prescribe me [controlled substance – sedative] or similar to help with anxiety/sleeplessness.

Portal user

Some patients noted how stress brought on by the circumstances of the hurricane resulted in physiological responses:

The stress of this situation has induced a few panic attacks. I realize I am due for an office visit but it is not possible during this flooding situation and caring for my elderly mother. I would appreciate a one month renewal on these medications until we get this situation squared away.

Portal user

Others conveyed descriptive accounts of their own circumstances during Hurricane Harvey that led to feelings of reduced mental health and well-being when making prescription requests:

...Unfortunately, [we] (our 6 year-old son)...were not so lucky. We took on serious amounts of water/sewage throughout our entire home and ended up having to make a treacherous emergency evacuation walking in water above our chest (with my son on my head) for 90 minutes through rushing currents to reach safety...The stress of worrying about my son and [name] (who slipped under the water and was sucked down [from] the undercurrent) is leaving me completely anxious and unable to calm down...

Portal user

Some users asked to be connected directly with a mental health professional through the portal during the disaster. This situation was addressed by providers sending referrals through the portal:

...this catastrophe...likely caused blackout[s] for people who suffered in past. Do you want to see therapist or psychiatrist, [so] I can place correct order[?]

Provider

#### Technical Difficulties

Patients’ messages described communication difficulties that were often related to office closures, phone malfunctions, and other crisis-related technical difficulties. Some patients said that they wanted to call an office to confirm their appointment or check if the office was open, but the office phone was not working:

We have tried calling the [location] office, but the recording says that the line is not in service. We’ve also tried calling the [alternative location] office number but could not get through. Please advice [sic].

Portal user

Several messages reflected that patients’ prescriptions were not delivered to the pharmacy because of a problem with the pharmacy’s electronic system. One provider messaged to inform a patient of the circumstance:

We have noticed in our office that since the storm some electronic prescriptions are not going through, I am guessing due to damaged fax lines and EMR malfunctions. Please let us know if your prescription did not transmit and we can call it in.

Provider

Some patients mentioned that communication was delayed because internet access was limited on account of Hurricane Harvey. Other patients messaged to report difficulties in rescheduling an appointment or surgery because of an issue in the web-based scheduling system:

I tried to re-schedule my appt from...yesterday for Fri 09/01, at 2:00. Online scheduling showed that date/time available but now it doesn’t show as scheduled...I have no idea whether I should come in Friday at 2:00 or schedule another time or?

Portal user

#### Provider Constraints

Occasionally, in their responses to patients, providers noted their own difficulties in fully addressing patient needs through the portal during the disaster. The providers mentioned technical complications, including loss of power and internet access, which delayed message responses; flooding that inhibited their ability to travel to medical facilities; technical issues related to accessing the EHR; office closure due to flooding; and inadequate ability to assess some medical conditions through a digital portal. Many complications in addressing patient messages were related to providers’ home or office damage (eg, flooding and loss of power) caused by the storm. Further obstacles were noted due to inadequate access to patient records through the EHR or limited to no access to the internet and patient portal to readily respond to patients’ requests:

Sorry I did not answer you sooner - we did not have any electricity or internet so I could not get on [the patient portal]. Please call the oncall number if you have any problems - they will contact me or another post coordinator to assist...

Provider

We found that while providers were navigating storm-related issues, other members of the health care team were able to alert patients regarding delayed provider responses:

Good Morning, I apologize for the inconvenience upon you at this time. Dr. [X] is not available like others he is unable to return to work at this time, but he is checking his messages throughout the day. When he responds I will definitely respond to this message.

Provider

Additional complications prevented providers from adequately addressing patient concerns through the portal. Certain conditions must be evaluated in office; thus, some providers noted that the digital portal was not sufficient and referred patients to urgent care centers with guidance as appropriate:

Dr. [X] is not able to access [electronic health record software] at this time, and the office is closed Monday and Tuesday. If your symptoms [s]ignificantly worsen or anything changes, go to urgent care...

Provider

For additional representative quotations in each of the themes discussed above, see Textbox 1.

**Representative quotes of qualitative portal message themes. All quotes are from patient portal users, unless otherwise noted.**

**Access**

- “I am sorry but I will have to postpone my today’s appointment due to Hurricane Harvey and associated rain and traffic mess on Houston roads. I will reschedule after next week once everything calms down.”
- “Due to the storm, my appointment with the Orthopedic surgeon got cancelled...I don’t know for how long. Can you refer me to another Doctor as I don’t want to keep waiting...in case my foot is not healing properly?”

**Prescription requests**

- “I have been flooded out of my house and don’t have any of my meds. Can you call in to [my pharmacy location] with a few days of each of my meds?”
- “Sunday I was rescued by boat from my home and a holed up now at [location]. – also lost both vehicles in the flood. Really stressed out. Can you prescribe something to calm me through the next few weeks?”

**Medical advice**

- “...based on the condition with the weather would it be possible for me to go to urgent care to get my stitches remove, it is becoming increasingly uncomfortable.”
- “All week my eyes were fine while I was taking the steroids. Since yesterday they are starting to itch and the lids are red and swollen again. What would you suggest I do?...”

**Mental health**

- “Due to recent events, (Tropical Storm Harvey,...multiple family tragedies, moving,...etc), I am not feeling the effects from taking this medication. I was wondering if it was possible to at least temporarily increase the dosage or increase to twice daily?”
- “...my home was completely flooded I have lost all of my meds and breathing machine and inhalers I am having a hard time breathing and my anxiety is out the roof being in a shelter my stress is extremely high...please help me figure this out...”

**Technical difficulties**

- “I was away from my phone and did not answer in time. When I tried to call back, I get an error message...I am just wondering if my appointment is still schedule[d]...”
- “My [pharmacy] lost electricity and phones. They reopened today but still do not have phones. Please call in a script to [pharmacy] at [alternative location].”

**Provider constraints**

- “I did forward your message to Dr. [X], he is not able to get out his neighborhood. I hope to hear from him today on what labs he wants to order for you. I will let you know as soon as I put in the orders.” [provider]
- “Please call our office so one of our medical assistants can send your order. At this time I’m not able to fax anything, we are just working from a laptop and I’m currently in Dallas due to the storm.” [provider]

## Discussion

### Principal Findings

We found that prior use of the patient portal was highly predictive of both using the patient portal and sending a message during Hurricane Harvey. This study uniquely included encounter data as a proxy measure, which we expected to be predictive of portal use generally but also specifically during a disaster situation. More than half of the total number of portal users during Hurricane Harvey had an outpatient encounter within 4 weeks before or after the week of the hurricane. Patients with recent and upcoming interactions with the health system were also more likely to have used the portal and messaged providers during the main week of Hurricane Harvey, indicating the importance of including health care needs in portal use analyses.

Previous evidence during normal operations suggests that active portal users had higher comorbidity scores [36], and patients classified as high risk with comorbidities may benefit from using the patient portal messaging system [24,37-39], which extends to the importance of portal use during widespread natural disasters to address potentially urgent or serious medical care. Given that patients with health conditions who use secure electronic messaging may show an improvement in health outcomes [40,41], it could be a valuable extension of medical care to encourage patients with ongoing medical needs (eg, chronic comorbidities) to actively use the portal, particularly in emergency circumstances.

The significance of prior use lends support for portal training interventions or enrollment efforts to decrease racial, ethnic, and age disparities seen in the uptake of digital health tools [42,43]. A recent systematic review on barriers and facilitators to patient portal use noted the importance of prior use of the portal in combination with provider buy-in [44]. The Diffusion of Innovation Theory categorizes technology adoption based on time to adoption [45], which is influenced by the availability of, and familiarity with, technology [46] and relates to technology literacy and comfort with sharing health information [47]. Our results also showed a continued disparity in the use of the portal by race and ethnicity, with a higher likelihood of accessing the patient portal during Hurricane Harvey for White, non-Hispanic/Latinx users, which indicates utility in targeting populations classified as high risk and vulnerable for portal enrollment and training because portal use has been linked to increased patient engagement [48-50].

The overall message frequency decreased during the week of the hurricane, likely because of power outages, flooding, and infrastructure instability, but our qualitative message analysis indicated that patient portal messages have an important role to play in rapidly communicating the urgent health needs of patients to providers in situations where hospital or physician access becomes extremely limited. The analysis of such a large number of inquiries and responses sent during a disaster situation serves as a natural needs assessment of critical problems surrounding patients’ health and continuity of medical care during a disaster, including issues with care or medication access and technical and infrastructural issues caused by the storm. The portal uniquely allows providers the ability to address certain problems for their patients in the absence of routine operations during a disaster, which may potentially reduce the risk of treatment disruption for acute conditions (eg, postoperative care and disaster-related injuries) and management of chronic conditions (eg, diabetes, cancer, and hypertension), in addition to meeting or rerouting mental health needs as appropriate.

Although patient-provider communication was supported during the disaster through the portal, providers were unable to fully address all concerns in the messages sent by patients. In some cases, the medical advice sought was beyond a provider’s ability to give through web-based messaging, necessitating a rerouting of care or a specialist referral. Providers were sometimes unable to comply with medication requests made during Harvey because of technical difficulties and rules governing controlled substances. It is also notable that not all communication during a disaster directly relates to the crisis but highlights the necessity of maintaining health care continuity for the many patients with ongoing care needs. Examining the successful uses and acknowledging the limitations of the patient portal as it is currently positioned provide the basis for extending the portal functionalities to create an intentionally designed, disaster-prepared patient portal.

### Disaster-Prepared Portal Functionality

#### Key Logistical Suggestions

Proper disaster preparedness for health care is critical in areas where natural disasters, including hurricanes, blizzards, and wildfires, are frequent. Using the portal more readily in these scenarios could streamline communication but would likely require temporarily reducing restrictions to EHR access among health care providers within a given health care entity. Traditional workflows and contingency plans are typically inadequate during the rapidly evolving chaos of natural disasters. For example, cross-coverage teams for an entire facility or practice may not be able, or available, to communicate effectively with staff or patients. Facilitating access to patient information for broader cross-coverage and triage teams than would normally comprise an on-call or ride-out team is necessary to increase the pool of potential providers who have access to the EHR. Processes can be adopted to *precredential* qualified providers for access across practices or facilities in the event that this scenario occurs again. Doing so would allow a defined *disaster team* of providers, similar to on-call coverage, to respond to urgent needs through the patient portal, eliminating some of the delays we saw in communication during Harvey while reducing the burden of electronic communication systemwide for all clinicians during times of limited access. When operationalizing a disaster communication approach through the portal to include an expanded recipient list to enable rapid triaging of these messages in times of limited access, it should be noted that the implementation would likely not have a one-size-fits-all approach. On the basis of this study’s findings, we offer key logistical suggestions below to realize an intentionally designed, disaster-prepared patient portal.

#### Triaging and Rerouting Needs During Crisis Situations

Common portal requests during our study period included a wide range of medical advice, and the patient portal could be used to triage and appropriately redirect some of these needs to available sources of care, specifically now with the increase in use of telemedicine visits. A common practice for portal communications is to assign a dedicated triage nurse to serve as the messaging gatekeeper for patient portal communications [51]; this same principle could be used in the intentional design of disaster-prepared patient portals for crisis-related requests. During a disaster, messages could be automatically triaged based on patients’ responses to screening questions to direct or reroute patients to appropriate and available in-person visits or electronic consultations until normal care can resume. Using the patient portal for pressing care requests is not an intended use of the typical portal messaging system; however, as many of our messages reflected, this type of urgent request is likely to occur and increase in frequency in disaster situations.

Having an emergency-prepared portal that can triage patients based on need specifically in times of crisis is a means of preparing for times of disrupted health care access. For example, the use of patient portals to address mental health concerns has demonstrated positive impacts for patients [52,53], and providing guidance or redirection seems especially important for mental health–related needs during times of crisis. The patient portal could also have triaging questions specific to anxiety, stress, and mental health care (eg, “Would you like to speak to someone about your current level of stress?”) and provide information on the available mental health services. All emergency medical incidents should be rerouted to readily available behavioral health patient portal–certified care providers [54-58].

#### Automating Medication Refills

Several messages in our study were related to prescription medication requests, which further highlights the necessity of care continuity during a disaster and raises the question and feasibility of being able to automate select pharmacy requests through the portal. An unknown duration of postdisaster recovery leaves many patients without adequate quantities of medication for chronic and life-threatening conditions such as hypertension, diabetes, seizures, and cancer. Even in the presence of a disaster declaration policy that allowed pharmacists to prescribe a 30-day supply of medication without prescriber authorization [59], the volume of message content in our study pertaining to prescription refill requests suggests that either the requested medications fell outside the disaster declaration allowances (eg, schedule II medications, opioids, stimulants, and depressants) or that pharmacists, physicians, or patients were not aware of the policy. The portal is a means by which established patients can communicate their medication needs to their provider, and an intentionally designed disaster portal could enable a function to allow these patients to have refill requests be sent or redirected automatically to a new pharmacy or new location. Automating some of these requests using the functionality of the portal would provide a useful tool to patients in emergency situations where immediacy is heightened, thereby decreasing physician-required approvals during these disasters when providers too experience limited availability.

#### Prioritized Automated Rescheduling

Infrastructure deficiencies in telecommunication due to disaster damage may also hinder access to the standard pathways of medical communication. Rescheduling canceled appointments and procedures is a logistical challenge following days of closure because of a natural disaster. The patients’ messages expressed concern regarding the delay of their scheduled appointments and the inability to reach providers through landline phones because of widespread infrastructure damage in the Houston area. The portal could be used to allow patients who had appointment cancelations to reschedule at their convenience once the health system has established providers’ availability and return to work. This may be particularly useful for urgent (nonemergent) surgical procedures so that patients can make informed decisions on when they would be able to receive treatment versus pursuing alternative treatment locations. The feasibility and implementation of automating this rescheduling feature for a prioritized listing of patients based on unavoidable disaster delays would be a topic for an important future study to extend current portal functionalities. Although patients would likely benefit from a prioritized automated rescheduling patient portal functionality for disaster use, appropriate conditions must be in place to allow a smooth transition between normal and disaster operations.

### Implications for Portal Use in Future Disasters

The 2020 hurricane season was uniquely challenging in that the highest number of storms on record—30 storms [60]—formed, in addition to the beginning of the COVID-19 global pandemic that occurred in the months before the start of the hurricane season. These large-scale disasters occurring simultaneously created unprecedented, long-term health care challenges. As cities and states across the United States reached peak COVID-19 cases and hospitalizations, health care systems relied on public health guidance to limit in-person patient appointments and services [61,62]. There has been a large increase in the adoption of the patient portal in terms of general use and depth of use [63], including substantial dependence on the use of telehealth platforms and portal messaging for care management of patients [61,64]. Medical information technology allowed health care providers a key method with which to continue accommodating patient services safely while reserving hospital capacity for the most critically ill. Although the time frames of these disasters differ, inferences for health management can be drawn from both disaster situations to inform how the patient portal can facilitate health care during ongoing and future disaster operations.

Notably, singular disaster events such as Hurricane Harvey resulted in a short-term decrease in the overall frequency of patient portal messages and portal features, whereas the COVID-19 pandemic has necessitated a long-term demand for tools and platforms supporting virtual care (eg, telehealth or virtual visits and messaging through portals or other technology) that have increased the adoption of digital health tools. Both disasters illustrate the inherent value of information technology tools in providing quality care conventions that are typically attributed to direct, in-person patient-provider interactions. In addition, electronic consultations offer the benefit of disease containment and increased access to care, specifically for patients living in rural areas, and can take place without increasing time or travel demands for patients, pivotal for natural disasters, while limiting human contact and potential virus spread during this pandemic period.

### Limitations

This study includes several limitations. First, this study was conducted at a single health care system; therefore, the results have limited generalizability because portal use may differ by region. However, because the study site is located in a large, demographically diverse urban city, these findings may be applicable to other health systems to support the integration of preparedness and medicine during emergency events. Second, we noted that there were some messages where a proxy user (eg, an adult family member messaging on behalf of a patient) self-identified in the message; however, we were unable to verify proxy users through portal accounts because this variable was not a component of our data set. Third, the comorbidity data were based on discharge disposition, which accompanies all encounter types; however, not all patients had an encounter in 2017. Thus, it is possible that we have underrepresented the incidence of the disease. The comorbidity data were not included in the final multivariate models for this reason; instead, we used encounter data, realizing that although it is not a replacement for controlling for disease severity, there is likely some correlation. Fourth, we were unable to control for, or examine, differential portal use by payer type but suggest future researchers do so. Finally, this study’s needs assessment relied on the analysis of retrospective data, which should be complemented by user-centered prospective methods of deriving functional requirements to ensure well-informed intentional design [65].

### Conclusions

The potential uses for the patient portal, classified and categorized into themes, lend support for the broader integration of patient portals as part of routine care coordination and management. Identifying patients’ health needs that were communicated through electronic patient portal messaging during an acute, widely destructive natural disaster that resulted in severe erosion of infrastructure and medical care is the first step in expanding the use of the portal for disaster health management. Future research should use this natural needs assessment and engage providers, informaticists, and data architects to expand the capabilities of the patient portal in disaster situations when normal care operations are limited and patient needs are extreme. Future research and practice should also consider how to use the portal for disaster planning relevant to chronic care and pregnancy. We raised considerations for disaster-specific elements of the portal interface based on patients’ met and unmet needs. Most importantly, enabling provider teams to respond to patient needs during disasters would alleviate delays when provider access is inhibited because of the circumstances of the disaster, which was demonstrated in some of our provider messages (eg, internet access, power outages, and flooding). Our study conveyed the need for immediate medical advice during a disaster period when infrastructure is severely debilitated and typical emergency care is nonoperational or not feasible because of safety concerns. The patient portal could serve as an intentional bridge during times of disaster when the typical workflow structure of health care disintegrates.

## Appendix

Multimedia Appendix 1 Descriptive statistics of the total 2017 population, the patient subgroups of interest, and a comparison between portal users and portal nonusers.

## Abbreviations

ED: emergency department
EHR: electronic health record
OR: odds ratio
SMD: standardized mean difference
