# Supplementary material for: Assessing Patient Needs During Natural Disasters: Mixed Methods Analysis of Portal Messages Sent During Hurricane Harvey
Source: J Med Internet Res. 2021 Sep 1;23(9):e31264. doi: 10.2196/31264 (PMC8444041; doi:10.2196/31264)
Supplement: Multimedia Appendix 1 [file jmir_v23i9e31264_app1.docx]

**Multimedia Appendix 1.** Descriptive statistics of the total 2017 population, the patient subgroups of interest, and a comparison between portal users and portal nonusers

|  | | Total (n=557,024) | Harvey messaging (n=4079) | Harvey portal use (n=31,737) | Portal users in 2017 (n=225,271) | Portal nonusers in 2017 (n=331,753) | Standardized mean difference |
| --- | --- | --- | --- | --- | --- | --- | --- |
| Age (years), mean (SD) | | 52.14 (18.23) | 53.97 (16.20) | 52.29 (16.53) | 51.02 (16.55) | 52.89 (19.25) | 0.104 |
| **Age (years), n (%)** | |  |  |  |  |  | 0.181 |
|  | 18-44 | 200,737 (36.04%) | 1204 (29.52%) | 10,991 (34.63%) | 83,604 (37.11%) | 117,133 (35.31%) |  |
|  | 45-64 | 196,581 (35.29%) | 1645 (40.33%) | 11,925 (37.57%) | 87,505 (38.84%) | 109,076 (32.88%) |  |
|  | ≥65 | 159,703 (28.67%) | 1230 (30.15%) | 8821 (27.79%) | 54,162 (24.04%) | 105,541 (31.81%) |  |
|  | Unknown | 3 (0.00%) |  |  | 0 (0.00%) | 3 (0.00%) |  |
| **Sex, n (%)** | |  |  |  |  |  | 0.084 |
|  | Female | 346,846 (62.27%) | 2723 (66.76%) | 20,737 (65.34%) | 145,730 (64.69%) | 201,116 (60.62%) |  |
|  | Male | 210,152 (37.73%) | 1356 (33.24%) | 11,000 (34.66%) | 79,537 (35.31%) | 130,615 (39.37%) |  |
|  | Unknown | 26 (0.00%) | 0 (0.00%) | 0 (0.00%) | 4 (0.00%) | 22 (0.01%) |  |
| **Race, n (%)** | |  |  |  |  |  | 0.264 |
|  | White | 363,204 (65.20%) | 3016 (73.94%) | 22,563 (71.09%) | 157,957 (70.12%) | 205,247 (61.87%) |  |
|  | Black | 97,358 (17.48%) | 447 (10.96%) | 3791 (11.95%) | 28,231 (12.53%) | 69,127 (20.84%) |  |
|  | Asian | 31,170 (5.60%) | 221 (5.42%) | 2151 (6.78%) | 15,480 (6.87%) | 15,690 (4.73%) |  |
|  | Other | 20,437 (3.67%) | 161 (3.95%) | 1340 (4.22%) | 8923 (3.96%) | 11,514 (3.47%) |  |
|  | Unknown | 44,855 (8.05%) | 234 (5.74%) | 1892 (5.96%) | 14,680 (6.52%) | 30,175 (9.10%) |  |
| **Ethnicity, n (%)** | |  |  |  |  |  | 0.205 |
|  | Non-Hispanic/Latinx | 450,016 (80.79%) | 3573 (87.59%) | 27,675 (87.20%) | 192,372 (85.40%) | 257,644 (77.66%) |  |
|  | Hispanic/Latinx | 77,022 (13.83%) | 372 (9.12%) | 2976 (9.38%) | 24,733 (10.98%) | 52,289 (15.76%) |  |
|  | Unknown | 29,986 (5.38%) | 134 (3.29%) | 1086 (3.42%) | 8166 (3.62%) | 21,820 (6.58%) |  |
| **Comorbidities, n (%)** | |  |  |  |  |  |  |
|  | Myocardial infarction | 8384 (1.51%) | 62 (1.52%) | 408 (1.29%) | 2444 (1.08%) | 5940 (1.79%) | 0.059 |
|  | Cancer | 15,310 (2.75%) | 204 (5.00%) | 1309 (4.12%) | 6907 (3.07%) | 8403 (2.53%) | 0.032 |
|  | Cerebrovascular disease | 10,985 (1.97%) | 115 (2.82%) | 688 (2.17%) | 3884 (1.72%) | 7101 (2.14%) | 0.030 |
|  | Chronic pulmonary disease | 27,948 (5.02%) | 282 (6.91%) | 1649 (5.20%) | 9439 (4.19%) | 18,509 (5.58%) | 0.064 |
|  | Congestive heart failure | 16,118 (2.89%) | 126 (3.09%) | 825 (2.60%) | 4616 (2.05%) | 11,502 (3.47%) | 0.087 |
|  | Connective tissue disease | 5688 (1.02%) | 90 (2.21%) | 482 (1.52%) | 2581 (1.15%) | 3107 (0.94%) | 0.021 |
|  | Diabetes without complications | 11,909 (2.14%) | 97 (2.38%) | 651 (2.05%) | 3296 (1.46%) | 8613 (2.60%) | 0.080 |
|  | Metastatic carcinoma | 3435 (0.62%) | 42 (1.03%) | 283 (0.89%) | 1400 (0.62%) | 2035 (0.61%) | 0.001 |
|  | Mild liver disease | 12,364 (2.22%) | 196 (4.81%) | 1243 (3.92%) | 5943 (2.64%) | 6421 (1.94%) | 0.047 |
|  | Moderate or severe liver disease | 1835 (0.33%) | 32 (0.78%) | 179 (0.56%) | 759 (0.34%) | 1076 (0.32%) | 0.002 |
|  | Peptic ulcer disease | 2229 (0.40%) | 36 (0.88%) | 159 (0.50%) | 872 (0.39%) | 1357 (0.41%) | 0.003 |
|  | Peripheral vascular disease | 16,147 (2.90%) | 198 (4.85%) | 1263 (3.98%) | 6268 (2.78%) | 9879 (2.98%) | 0.012 |
|  | Renal disease | 19,522 (3.50%) | 162 (3.97%) | 1165 (3.67%) | 6011 (2.67%) | 13,511 (4.07%) | 0.078 |
| **Encounter type, n (%)** | |  |  |  |  |  |  |
|  | Emergency department (ED) | 31,564 (14.01%) | 576 (14.12%) | 3677 (11.59%) | 31,564 (14.01%) |  |  |
|  | Inpatient | 24,159 (10.72%) | 635 (15.57%) | 3908 (12.31%) | 24,159 (10.72%) |  |  |
|  | Observations | 13,201 (5.86%) | 314 (7.70%) | 2064 (6.50%) | 13,201 (5.86%) |  |  |
|  | Outpatient | 86,139 (38.24%) | 2065 (50.63%) | 13,808 (43.51%) | 86,139 (38.24%) |  |  |
|  | Surgery | 24,210 (10.75%) | 615 (15.08%) | 4140 (13.04%) | 24,210 (10.75%) |  |  |
